# How the Cognitive Status of Older People Affects Their Care Dependency Level and Needs: A Cross-Sectional Study

**DOI:** 10.3390/ijerph191610257

**Published:** 2022-08-18

**Authors:** Halina Doroszkiewicz

**Affiliations:** Department of Geriatrics, Medical University of Bialystok, 15-471 Bialystok, Poland; halina.doroszkiewicz@umb.edu.pl

**Keywords:** geriatric assessment, care dependency, cognitive function, older adults

## Abstract

Introduction: The decline in health and abilities as a result of the aging process leads to a growing need for care and various forms of support. The aim of this study was to find out the level and the main areas of care dependency among older persons with cognitive impairment versus those without cognitive impairment. Materials and Methods: A cross-sectional study was conducted among 200 older persons hospitalized in the years 2017–2018 at a geriatric ward in Poland. The research took into consideration the socio-demographic variables of the older people (age, sex, marital status, mode of dwelling, health self-evaluation, and loneliness) and the results of the assessment of their functional status, including their physical functional status according to the Barthel scale and the I-ADL, locomotion, the risk of falls and pressure sores, emotional state, cognitive function status, vision, hearing, and the Polish version of the Care Dependency Scale. Results: People with cognitive impairment significantly more often have poorer results in regard to ADL and I-ADL physical functions, locomotion, risk of depression, falls, pressure sores, as well as hearing and vision problems, than people with good cognitive status. The results of the study indicate that the advancing impairment of cognitive functions in older people has an impact on the level of care dependency. Conclusion: The results of this original research show that persons with cognitive impairment are significantly more often dependent on external assistance in regard to all the needs assessed in the CDS than people without such impairment. The spectrum and number of needs in which the older person requires help grows significantly with the advancement of cognitive impairment. Older people with cognitive impairment who live alone require special support from formal caregivers in their home environment. Identification of the level of dependency and the needs of older people with cognitive impairment is of key importance for planning caregiving resources.

## 1. Introduction 

Currently, Poland sees a very dynamic process of population aging [1]. The decline in health and abilities as a result of the aging process leads to a growing need for care and various forms of support [2,3,4]. The group of older people, however, is not uniform, and its heterogeneity results from differences in the process of aging itself and in the chronic diseases that seniors may develop [5]. The proportion of older persons with physical impairments (assessed with tests referring to activities of daily living), emotional state deterioration (depression), and cognitive impairments (especially dementia), and fraility syndrome, grows with age [6,7,8]. In people over 65 years of age, cognitive impairments are a serious problem, leading to a decrease in functional capacity, quality of life, and autonomy, and a higher dependence on assistance from other people [8,9,10]. 

The results of research by PolSenior [11] carried out in the population of older people in Poland show an increase in the prevalence and advancement of cognitive deficit with age. On average, one in six persons aged 60 or over displayed cognitive deficits to the degree implying dementia. Cognitive deficit most often occurred in people aged 80 or over with the lowest level of education, making a population that is especially susceptible to late diagnosis and treatment in daily clinical practice [11]. Advancing deterioration of cognitive functions leads to poorer mental capacity, ability to perform the activities of daily living, and behavioral changes. It intensifies problems connected with dependence on other people’s assistance and forces the need to provide care [12,13]. 

Care dependency means that the person’s ability to carry out activities of daily living (taking a shower, preparing meals, taking medications, locomotion, managing money, recreation, or social contacts) decreases, and the person becomes partially dependent on other people’s help in satisfying their care needs [14]. Care dependency is a nursing concept defined by Dijkstra [15]. The concept is based on the assumption that a professional offers support to patients whose self-care abilities decreased and whose care needs make them somewhat dependent. The objective of support is to restore the patient’s autonomy in providing self-care [15]. Literature presents many causes of care dependency among older people [16]. The factors that may contribute to it are age, health status, functional disability, dementia, and frailty [16]. Older people’s growing dependence on care and the resulting nursing problems, such as pressure sores, immobility, incontinence, falls, and dementia are considered as the most frequent causes of transferring the person to an institution [17,18]. Apart from providing care, long-term care institutions monitor the quality of services they offer so as to stabilize or improve care and to counteract negative consequences, such as increased care dependency, reduced quality of life, or death [18].

The results of research carried out among patients of geriatric wards and residents of nursing homes confirm the influence of age and dementia as the main factors causing dependence on other people’s help [18]. Older people were highly dependent on care in aspects such as hygiene, mobility, learning ability, avoidance of danger, recreational activity, and their sense of rules and values [19]. The authors of the research show that signs of poorer cognitive and physical functioning appear particularly in the last phase of life, which confirms the assumption that care dependency in that period is high [19].

Patients with dementia are an especially vulnerable group because of communication difficulties, even to the point of being unable to express their needs, as well as co-occurring behavioral problems, which often scare and exhaust family caregivers [20]. Coping with these problems becomes the main task for families and formal caregivers [20]. Therefore, it is necessary to gain more insight into dependency, its dynamics, and the spectrum of needs of people with cognitive impairments admitted to hospitals and long-term care institutions. This could help service providers organize and ensure more effective, suitable care, and in the long run, improve quality of life. In Poland, there is a lack of research on this subject, and the research presented in this article is an attempt to, at least partially, fill the gap.

The aim of this study was to find out the level and the main areas of care dependency among older persons with cognitive impairment versus those without cognitive impairment.

Specific aims of the study were:

1. To evaluate the functional status of older people with cognitive impairment versus those without cognitive impairment.
2. To evaluate the degree and aspects of dependency of older people with cognitive impairment versus those without cognitive impairment.

## 2. Materials and Methods

### 2.1. Participants and Setting 

The research covered 200 older persons hospitalized in the years 2017–2018 at a geriatric ward in Poland. It was a secondary analysis of data collected during the cross-sectional study on health problems, and using the Care Dependency Scale for predicting care needs among geriatric patients of advanced age [21]. Older people with many diseases and physical and/or cognitive dysfunction were referred to the geriatric ward by general practitioners and were mainly admitted in a planned manner. During their stay in the ward, every older person undergoes a comprehensive geriatric assessment (CGA). CGA is performed routinely at the ward by an interdisciplinary team of a physician, a nurse, a physiotherapist, and a psychologist [22]. CGA is a multidimensional and multidisciplinary diagnostic process. Its aim is to identify the causes of the deterioration of functions, planning and/ or delivering care, and diagnosing geriatric syndromes [22]. We confirm that all participants consented to participate in the study by means of written informed consent. The study was carried out in accordance with the Declaration of Helsinki and approved by the Bioethics Committee of the Medical University of Bialystok (Resolution APK. 002.194.2022).

### 2.2. Measurements and Procedure

The research took into consideration the socio-demographic variables of the older people (age, sex, marital status, and mode of dwelling: alone, with a mate, with children, in an institution, health self-evaluation, and loneliness) and the results of the assessment of their functional status (Table 1), including physical functional status according to the Barthel scale and the I-ADL, locomotion, risk of falls and pressure sores, emotional state, cognitive function status, vision, and hearing.

The physical functional status measured with the Barthel scale shows the older person’s ability to carry out basic activities of daily living (ADL): locomotion, eating and drinking, using the toilet, hygiene, washing, bathing, mobility, climbing up and down the stairs, getting dressed and undressed, and urinary and fecal continence. The total score may range from 0 points (complete dependency) to max. 100 points (complete independence) [23].

Functional status involving instrumental activities of daily living (I-ADL) was evaluated using the Duke OARS assessment [24]. Six domains of instrumental functions were assessed (washing the floor and other cleaning work, using the phone, preparing meals, doing the shopping, managing money, and taking medications). The total score ranges from 0 points (the lowest function) to 12 points (the highest function).

Balance and gait were assessed using the Performance Oriented Mobility Assessment (POMA) test. The score of 0 points shows the inability to perform any task, and the maximum score of 10 points reflects complete independence [25].

The risk of pressure sores was assessed using the Norton scale, where the score of ≤14 points shows there is a risk of developing pressure sores [26].

The older people’s emotional state was evaluated using Yesavage’s Geriatric Depression Scale (GDS), in which the range of 0–5 means no depression, and 6–15, a growing risk of depression [27].

The questionnaires used the research of the Polish version of the Care Dependency Scale (CDS) [28]. The CDS can be used to assess 15 biopsychosocial needs a human naturally has and needs to satisfy, whether healthy or ill. A five-point Likert scale is used in the evaluation, with 1 meaning complete dependency on other peoples’ care, 2 meaning high dependency, 3 meaning partial dependency, 4 meaning limited dependency, and 5 meaning the patient is almost independent of external care. The interpretation of the scores in the scale makes it possible to assign each patient to one of three levels of dependency: the 15–44 range means a high level of care dependency, 45–59, medium dependency, and 60–75, low dependency.

The cognitive status was evaluated using the Abbreviated Mental Test Score by Hodgkinson (AMTS) [29]. The participants were divided into the following three groups on the basis of cognitive status assessment, where the range of 0–3 points means severe impairment of cognitive functions, 4–6 means moderate impairment, and 7–10 means normal cognitive status. The division into the three study groups is presented in Table 1.

### 2.3. Statistical Analysis

The results were subject to statistical analysis, i.e., calculating the arithmetic mean and standard deviation for quantitative variables, and the quantitative and percentage distribution for qualitative variables. In order to compare the distribution of variables between the groups, Pearson’s chi-squared test was used with the value of *p* < 0.05 considered as statistically significant. The Mann–Whitney U test was used in the case of measurable characteristics for two independent samples, and the Kruskal–Wallis test was used for a higher number of samples. The statistical analysis was carried out with the use of the STATISTICA 13.0 package (StatSoft, Tulsa, OK, USA). 

## 3. Results

Table 1 shows the socio-demographic characteristics and the results of the functional assessment of older people in three groups of cognitive status. The average age of the participants was 80 years and over and was significantly higher in the group with severe cognitive impairment: 83.6 years old, *p* = 0.011. The vast majority of participants were widowed women; their proportion was similar in all the compared groups. Dwelling alone was slightly more frequently observed in the group of people with cognitive impairment as compared to the group without cognitive impairment. Persons with impaired cognitive functions felt lonely significantly more often than persons without cognitive impairment, *p* = 0.087. Individuals with cognitive impairment considered their health as poor or very poor significantly more often than those without cognitive impairment, *p* = 0.007.

The results of the functional status assessment show statistically significant differences between the groups. Poor functional status was significantly more often displayed by persons from the group with severe cognitive impairment than by persons without cognitive impairment in regard to the following functions: locomotion (83.7% vs. 65.3%), the risk of falling (75.6% vs. 48.0), hearing problems (53.1% vs. 40.8%), and vision problems (42.9% vs. 37.8).

Evaluation of the participants’ physical status in terms of the ability to perform basic and instrumental activities of daily living (ADL and I-ADL) showed statistically significant differences between the groups. People with cognitive impairment displayed poorer performance in regard to the assessed physical functions (ADL and I-ADL) than people without cognitive impairment (data in Table 1).

The risk of depression (7.3 vs. 5.3) and the risk of pressure sores (15.4 vs. 17.3) were observed significantly more often in people with cognitive impairment than in people without the impairment (data in Table 1).

Further, the level of care dependency according to the CDS was assessed in each group of cognitive function status (data in Table 2).

The average CDS index in the study groups was: severe cognitive impairment—46.3 (±16.1), moderate cognitive impairment—53.6 (±14.3), and no cognitive impairment—60.8 (±12.6), respectively. The differences were statistically significant, *p* = 0.000 (data in Table 2).

The level of care dependency was statistically significantly correlated with the participants’ cognitive status, *p* = 0.0001 (data in Table 2). The results show that the highest level of care dependency was observed among people with severe cognitive impairment in regard to satisfying the following needs: recreational activities (1.9), learning activities (2.0), daily activities (2.6), avoidance of danger (2.9), mobility (2.9), hygiene (3.1), day/night patterns (3.2), continence (3.2), contact with others (3.3), body temperature (3.3), eating and drinking (3.4), getting dressed and undressed (3.4), body posture (3.4), and sense of rules and values (3.6) (data in Table 2).

Next, the author analyzed the number of needs listed in the CDS in which the participants needed assistance. A higher number of needs was significantly more often observed in the group of persons with severe cognitive impairment than among those without cognitive impairment. This referred to: physical needs (4.7 vs. 2.3), *p* = 0.000, psychological needs (1.7 vs. 0.9), *p* = 0.000, social needs (1.7 vs. 0.6), *p* = 0.000, and the sum of biopsychosocial needs (8.0 vs. 3.8), *p* = 0.000 (data in Table 2).

**Figure 1:**
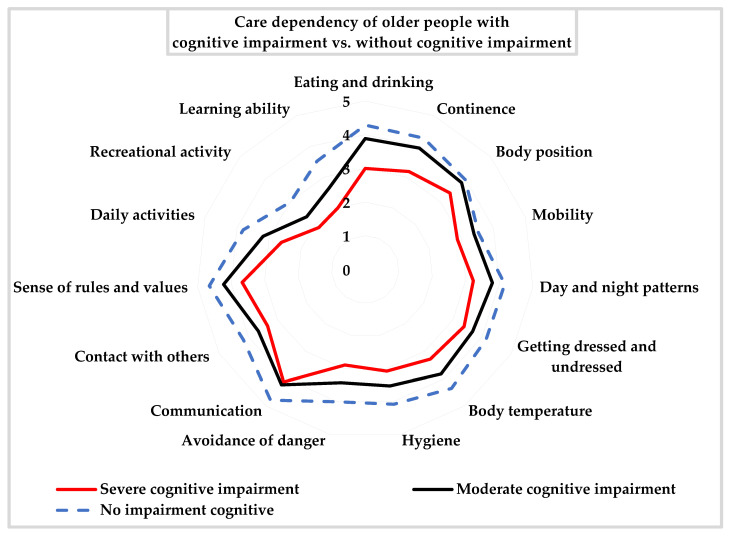
The level of severity of care dependency of older people with cognitive impairment vs. without cognitive impairment.

Figure 1 illustrates precisely the level of care dependency in different cognitive function groups.

## 4. Discussion

The aim of this research was to evaluate the functional status of older people and to compare the degree and main aspects of care dependency of persons with and without cognitive impairment. The analysis of age in the studied groups showed a significantly higher age of persons with cognitive impairment (83.6 vs. 80.6 years old). The results show that the vast majority of the participants were widowed women, with a similar proportion in the compared groups. Living alone and feeling lonely were slightly more frequently observed in the group of people with cognitive impairment. Poor or very poor health self-assessment was more often observed in the group of people with cognitive impairment. The analysis of the results of participants’ functional status assessment show differences between the compared groups. Persons with cognitive impairment, significantly more often, had a poorer physical functional status, i.e., the ability to perform basic (ADL) and complex (I-ADL) activities of daily living. They were significantly more often disabled in terms of locomotion and displayed an increased risk of pressure sores and problems with vision and hearing. The analysis of the care dependency level according to the CDS showed differences between the compared groups (differing in terms of cognitive status). The severity of dependency grew significantly with the advancement of cognitive impairment of the participants. The results of this original research show that persons with cognitive impairment were significantly more often dependent on external assistance in regard to all the needs assessed in the CDS than people without such impairment. 

The results of research by Schlüsler et al. [19] carried out among patients of nursing homes show that people with dementia were completely or highly dependent on external assistance. They were mostly dependent in terms of the following needs: day/night patterns, contact with others, sense of rules and values, and communications. The comparison of the dependency level between stages of dementia showed a difference between moderate and severe dementia (9.3% vs. 44.3% individuals completely dependent on other people’s care). 

The results of a study by Kim [30], carried out among 110 demented residents of long-term care institutions in Korea, show that care dependency was significantly influenced by factors such as cognitive functions, functional disability, and dementia. The results of studies by other authors [31], carried out among patients of nursing homes with moderate to severe dementia, show prognostic factors contributing to dependence on external help, such as cognitive and physical abilities and apathy. Other authors’ research conducted among patients with dementia staying at care institutions showed that care dependency significantly depends on cognitive functions, functional disability, behavioral dysfunction, and the duration of dementia [32].

The original study showed that the spectrum and number of needs in which the older person requires help grows significantly with the advancement of cognitive impairment. Care dependency mostly referred to the following aspects: recreational activities, learning activities, daily activities, avoidance of danger, mobility, hygiene, day/night patterns, continence, contact with others, body temperature, eating and drinking, getting dressed and undressed, body posture, and sense of rules and values.

A study carried out by other authors among people with dementia living at home, at risk of institutionalization, and recently institutionalized, confirmed the relationship between dementia and the kind and degree of care dependency [33]. It confirmed a higher level of dependency in persons staying in care institutions than in persons receiving care at home with respect to needs such as using the toilet, getting dressed and undressed, and incontinence [33]. A study conducted in Portugal among 329 persons with cognitive impairment showed that individuals with dementia staying in long-term care institutions had serious mobility limitations [34].

The study may help service providers and informal caregivers providing care for older people at home to gain some insight into the spectrum of biopsychosocial needs and the care dependency levels with respect to the progression of cognitive impairment. The results of the analyses carried out among older people with cognitive impairment may help to better understand the needs, who needs special care and attention due to advancing memory disorders, communication difficulties, decreasing self-care abilities, and a growing risk of nursing problems (pressure sores, urinal incontinence, falls, or mobility problems). Furthermore, the results of original research reveal specific needs of people with cognitive impairment in which care dependency develops to the greatest extent, such as recreational activities, learning activities, daily activities, avoidance of danger, and mobility. The research results may also help care organizers better adjust the care plan to the patient and implement prevention measures (i.e., the use of facilities, mobile equipment, and other forms of assistance) in order to improve and/or stabilize the level of dependency, as well as minimize nursing problems. 

Regular evaluation, i.e., monitoring the needs and functional status of people with cognitive impairment, may help properly organize adequately addressed care, prevent the progression of dependency and premature institutionalization, improve the quality of life, and rationalize the costs of care. 

### Limitations of the Study

The study has some limitations. The cross-sectional character of the study does not allow for statistical inference as to the causal relationships between the variables. The small size of the sample of people with cognitive impairment vs. those without the impairment only made it possible to differentiate very well between the variables concerning the assessment of functional status, level of dependency, and the main aspects of the needs in the studied groups. The relationships between particular characteristics were confirmed using statistical methods. 

## 5. Conclusions 

The results of the original study indicate that advancing impairment of cognitive functions in older people has an impact on their level of care dependency. People with cognitive impairment significantly more often have poorer results in regard to ADL and I-ADL physical functions, locomotion, risk of depression, falls, pressure sores, as well as hearing and vision problems, than people with good cognitive status. The severity of care dependency and the spectrum and number of needs in which the person requires help grow significantly with the advancement of cognitive impairment. Care dependency mostly refers to the following aspects: recreational activities, learning activities, daily activities, avoidance of danger, mobility, hygiene, day/night patterns, continence, contact with others, body temperature, eating and drinking, getting dressed and undressed, body posture, and sense of rules and values. 

Older people with cognitive impairment who live alone require special support from formal caregivers in their home environment. 

Insight into the natural course of care dependency in people with cognitive impairment is necessary to organize and optimize individual care adjusted to the patient’s needs. Identification of the level of dependency and needs of older people with cognitive impairment is of key importance for planning caregiving resources. 

## Figures and Tables

**Table 1:** Socio-demographic characteristics and the results of functional status assessment in different cognitive function groups (*n* = 200).

Characteristics	Severe Cognitive Impairment	Moderate Cognitive Impairment	No Impairment	*p*-Value
	*n* = 49	*n* = 53	*n* = 98	
Age in years				
Mean ± SD	83.6 ± 5.9	82.3 ± 6.0	80.6 ± 7.0	0.011
Min.	66	68	65	
Max.	92	93	101	
Female (%)	61.2	79.2	67.3	0.128
Marital status (%)				
married	45.8	28.6	32.4	0.310
widowed	54.2	69.1	60.8	
divorced	0.0	2.3	6.8	
Dwelling (%)				
alone				
with a mate	35.4	47.6	33.3	
with children	41.7	21.4	30.7	0.333
in an institution	22.9	31	32	
	0.0	0.0	4.0	
Does the person feel lonely?				
often	63.2	84.9	65.3	0.087
Health self-assessment (%)				
very good/good	24.5	45.3	50.0	0.007
poor/very poor	75.5	54.7	50.0	
Problem with locomotion (%)	83.7	62.3	65.3	0.001
Risk of fall (%)	75.6	56.6	48.0	0.019
Use of glasses (yes) (%)	42.9	41.5	37.8	0.930
Use of hearing aid (yes) (%)	53.1	37.8	40.8	0.207
Barthel Index, mean ± SD				
(0–100)	67.7 ± 30.2	75.8 ± 26.6	82.3 ± 23.6	0.001
I-ADL,				
mean ± SD (0–12)	4.7 ± 3.4	5.8 ± 3.9	7.6 ± 3.4	0.000
GDS,				
mean ± SD (0–15)	7.3 ± 3.7	6.3 ± 3.6	5.3 ± 3.7	0.013
AMTS,				
mean ± SD (0–10)	2.3 ± 0.8	5.4 ± 0.8	8.8 ± 1.1	0
Norton scale, mean ± SD (0–20)	15.4 ± 3.3	16.3 ± 3.1	17.3 ± 2.7	0.001

Note: The *p*-value reflects the significance of the listed characteristics in particular groups. Abbreviations: Min.—minimum, Max.—maximum, I-ADL—Instrumental Activities of Daily Living, GDS—Geriatric Depression Scale, AMTS—Abbreviated Mental Test Scoring, CDS—Care Dependency Scale. The Mann–Whitney U test, the Kruskal–Wallis test, and the *p*-value for Pearson’s chi-squared test/Fisher’s exact test were used when comparing the groups.

**Table 2:** The level of care dependency and care needs in groups of different cognitive status (*n* = 200).

Characteristics	Severe Cognitive Impairment	Moderate Cognitive Impairment	No Impairment	*p*-Value
	*n* = 49	*n* = 53	*n* = 98	
Care Dependency Scale,				
mean ± SD (15–75)	46.3 (±16.1)	53.6 (±14.3)	60.8 (±12.6)	0.0000
Severity level of care dependency,				
*n* (%)				
1 = completely care dependent	6 (12.2)	3 (5.7)	1 (1.0)	0.0001
2 = to a great extent care dependent	15 (30.6)	10 (18.9)	13 (13.3)	
3 = partially care dependent	17 (34.8)	18 (34.0)	17 (17.4)	
4 = to a limited extent care dependent	5 (10.2)	17 (32.0)	39 (39.7)	
5 = almost independent	6 (12.2)	5 (9.4)	28 (28.6)	
Care Dependency Scale,				
mean ± SD				
Eating and drinking	3.4 ± 1.5	3.9 ± 1.3	4.3 ± 1.1	0.0004
mean ± SD (1–5)				
Continence, mean ± SD (1–5)	3.2 ± 1.4	3.9 ± 1.3	4.3 ± 1.2	0.0000
Body posture, mean ± SD (1–5)	3.4 ± 1.3	3.8 ± 1.1	4.0 ± 1.1	0.0129
Mobility, mean ± SD (1–5)	2.9 ± 1.4	3.4 ± 1.4	3.5 ± 1.3	0.0248
Day/night pattern, mean ± SD (1–5)	3.2 ± 1.4	3.8 ± 1.1	4.2 ± 1.0	0.0001
Getting dressed and undressed, mean ± SD (1–5)	3.4 ± 1.4	3.7 ± 1.3	4.1 ± 1.2	0.0009
Body temperature,	3.3 ± 1.2	3.8 ± 1.2	4.4 ± 0.9	0.0000
mean ± SD (1–5)				
Hygiene, mean ± SD (1–5)	3.1 ± 1.5	3.5 ± 1.3	4.1 ± 1.2	0.0001
Avoidance of danger,	2.9 ± 1.3	3.4 ± 1.3	4.0 ± 1.2	0.0000
mean ± SD (1–5)				
Communications, mean ± SD (1–5)	4.1 ± 1.0	4.2 ± 1.0	4.8 ± 0.5	0.0000
Contact with others, mean ± SD (1–5)	3.3 ± 1.3	3.7 ± 1.3	4.2 ± 1.1	0.0004
Sense of rules and values, mean ± SD (1–5)	3.6 ± 1.4	4.2 ± 0.9	4.7 ± 0.7	0.0000
Daily activities, mean ± SD (1–5)	2.6 ± 1.3	3.2 ± 1.2	3.8 ± 1.3	0.0000
Recreational activities, mean ± SD (1–5)	1.9 ± 1.3	2.3 ± 1.5	3.0 ± 1.6	0.0001
Learning activities, mean ± SD (1–5)	2.0 ± 1.0	2.6 ± 1.1	3.5 ± 1.0	0.0000
Number of care needs CDS, mean ± SD				
Physical care needs	4.7 ± 3.2	3.2 ± 3.2	2.3 ± 2.9	0.0000
Psychological care needs	1.7 ± 0.7	1.5 ± 0.7	0.9 ± 0.8	0.0000
Social care needs	1.7 ± 1.5	1.2 ± 1.4	0.6 ± 1.0	0.0000
Sum score of biopsychological care needs, CDS, mean ± SD	8.0 ± 4.9	5.9 ± 4.8	3.8 ± 4.3	0.0000

## Data Availability

The data presented in this study are available on request from the author.
